# High prevalence of symptomatic spinal stenosis in Norwegian adults with achondroplasia: a population-based study

**DOI:** 10.1186/s13023-020-01397-6

**Published:** 2020-05-25

**Authors:** Svein O. Fredwall, Unni Steen, Olga de Vries, Cecilie F. Rustad, Heidi Beate Eggesbø, Harald Weedon-Fekjær, Ingeborg B. Lidal, Ravi Savarirayan, Grethe Månum

**Affiliations:** 1 Sunnaas Rehabilitation Hospital, TRS National Resource Centre for Rare Disorders, Nesodden, Norway; 2 Faculty of Medicine, Institute of Clinical Medicine, University of Oslo, Oslo, Norway; 3 Oslo University Hospital, Department of Medical Genetics, Oslo, Norway; 4 Oslo University Hospital, Division of Radiology and Nuclear Medicine, Oslo, Norway; 5 Oslo Centre for Biostatistics and Epidemiology, Research Support Service, Oslo University Hospital, Oslo, Norway; 6 Murdoch Children’s Research Institute and University of Melbourne, Parkville, Australia; 7 Sunnaas Rehabilitation Hospital, Department of Research, Nesodden, Norway

**Keywords:** Achondroplasia, Activities of daily living, Adults, Hand strength, 6-minute walk test, Spinal stenosis, Pain

## Abstract

**Background:**

Symptomatic spinal stenosis (SSS) is a well-known medical complication in achondroplasia. The reported prevalence of SSS is 10 to 30%, an estimate based on small studies or selected populations. No population-based studies exist currently. Furthermore, the relationship between SSS and physical functioning has not been investigated in detail. The aims of this study were to describe the prevalence of SSS in Norwegian adults with achondroplasia, and to explore the impact of SSS on physical functioning.

**Methods:**

This was a population-based study on Norwegian community-dwelling adults with genetically confirmed achondroplasia. Prevalence of SSS was defined by clinical symptoms, and confirmed by imaging or surgical reports. Physical functioning was assessed by walking capacity (6-min walk test), hand strength (Grippit), and activities of daily living (the Health Assessment Questionnaire, HAQ). Pain was assessed by pain site locations and intensity (Numeric Rating Scale, NRS).

**Results:**

In total, 50 participants were included (27 males, 23 females). Median age was 41 years (range 16 to 87 years), 34 (68%) had SSS. The estimated median age at first symptom onset was 33 years (95% confidence interval (CI) 29 to 43 years), range 10 to 67 years. The majority had multiple spinal levels affected. The walking distance was 110 m shorter in the SSS group (95% CI − 172 to − 40 m) as compared with the non-SSS group (*p* < 0.01). There was no considerable difference in hand strength between the two groups. Mean HAQ scores (0–3) for walking and hygiene were significantly higher in the SSS group, reflecting more activity limitations. Mean differences were 0.9 (95% CI 0.3 to 1.4, *p* < 0.01) and 0.6 (95% CI 0.2 to 1.0, p < 0.01). Pain intensity (NRS 0–10) was also significantly higher in the SSS group with a mean difference of 3.2 (95% CI 0.6 to 5.6, *p* = 0.02).

**Conclusions:**

SSS was highly prevalent in Norwegian adults with achondroplasia, with symptom onset at young age, and multiple spinal levels affected. The presence of SSS was associated with reduced walking distance, activity limitations, and more pain. The findings underline the importance of thorough assessment and monitoring of SSS in achondroplasia, including a formal assessment of physical functioning.

## Background

Achondroplasia is the most common skeletal dysplasia leading to disproportionate short stature. The condition is caused by a mutation in the gene coding for the Fibroblast Growth Factor Receptor 3 (*FGFR3*), and results in reduced proliferation and maturation of growth plate chondrocytes [1]. In addition to decreased bone elongation, the spinal canal diameter is reduced up to 50%, predisposing for symptomatic spinal stenosis (SSS) [2–4]. Characteristic symptoms of SSS are back pain and/or radiating pain into the buttocks or the legs, exacerbated by prolonged walking, standing or lumbar extension, and resulting in decreased walking distance [3–7]. Rest, lumbar flexion or squatting typically relieves symptoms [4, 6, 8]. Bladder and bowel symptoms can also occur [3, 7].

SSS is a well-known medical complication in adults with achondroplasia [4, 9, 10]. The prevalence has been reported to be between 10 and 30% [10–13]. However, these estimates were based on few or selected populations, and the methods for defining SSS were not described. We have not found prevalence rates reported in unselected achondroplasia populations [14], and our clinical experience indicates that the prevalence of SSS is much higher. Several studies have also found a decline in physical health in individuals with achondroplasia during the fourth decade, including a high prevalence of pain [15–18]. However, the relationship between SSS and physical functioning has not been investigated in detail.

The main objectives of this clinical study were to describe the prevalence of SSS in Norwegian adults with achondroplasia, and to explore the impact of SSS on physical functioning.

## Methods

### Study design

This clinical, cross-sectional study also included historical data. The study was part of the Norwegian Adult Achondroplasia Study, conducted between 2017 and 2019, which aimed to include as many of the total Norwegian adult achondroplasia population as possible.

### Patient and public involvement

The Norwegian Restricted Growth Association has been involved in initiating and planning the Norwegian Adult Achondroplasia Study. A group of six adults with achondroplasia cooperated in developing the study protocol, selecting main topics to be investigated, and piloting the questionnaires. A reference group has been established, consisting of two adults with achondroplasia and four healthcare professionals. The study has been conducted in accordance with the STROBE guidelines for reporting of observational studies [19].

### Recruitment

A written invitation was sent to all individuals aged 16 years or older who are registered with achondroplasia in the National Resource Centre for Rare Disorders’ database [20]. Invitations were also sent to individuals registered with achondroplasia at the University Hospitals of Norway’s four regional health trusts. A written reminder was sent after four to six weeks. The study was announced on the websites of the Resource Centre and The Norwegian Restricted Growth Association (NiK), using text and short videos for recruitment. Recruitment videos were also published on YouTube and Facebook. We informed about the study at the NiK summer gatherings in 2017 and 2018, as well as to other relevant institutions likely to meet adults with achondroplasia.

### Inclusion and exclusion criteria

Inclusion criteria were residents of Norway, aged 16 years or older, with a genetically confirmed diagnosis of achondroplasia, who spoke and understood the Norwegian language. Exclusion criteria were presence of severe cognitive deficits, mental illness, substance abuse or having a medical condition making them unable to participate in the study.

### Definition of symptomatic spinal stenosis (SSS)

This study defined SSS as the presence of, or history of, clinical symptoms of neurogenic claudication, radicular pain or both, *in combination with* spinal stenosis described at the correlating spine level in imaging reports or noted intraoperatively and described in the spine surgery records [6, 21]. Neurogenic claudication was defined as pain or discomfort that radiates beyond the spinal area into the buttocks and/or into the thighs or lower legs, are exacerbated by prolonged walking, standing or lumbar extension, and improved by rest or lumbar flexion [5, 6, 21]. To confirm the presence of spinal stenosis in symptomatic non-operated participants, the magnetic resonance images (MRIs) were collected and re-interpreted by an experienced radiologist. A cross-sectional anteroposterior spinal canal with a diameter of ≤10 mm at minimum one spine level was considered diagnostic of SSS [22–24].

### Data collection

The data collection process took place from March 2017 through March 2019. An experienced team, consisting of a medical doctor (SOF), physiotherapist (OdV), and occupational therapist (US), extracted data from medical records and performed the structured clinical examinations, interviews, and physical tests, according to a predefined study protocol. All the investigations were conducted during a 2.5-day stay at Sunnaas Rehabilitation Hospital. Five participants were not able to come to the hospital, due to impaired health, and were interviewed and examined during a home visit by one of the authors (SOF).

#### Demographic and clinical data

Demographic information was obtained by a self-administrated, custom-made questionnaire, and verified by a clinical interview. A detailed medical history regarding symptoms of spinal stenosis was obtained, and a clinical neurological examination was performed on all participants. Abnormal neurological findings were defined as absent reflexes (grade 0) or hyperreflexia (3+ or clonus), impaired sensation (0 or 1 tested with a wisp of cotton and single-use pin), or reduced muscle strength (grade 0 to 3 on a manual muscle strength testing scale from 0 to 5) [25]. Reported first symptom onset was based on information given by the participants, and confirmed by the medical records. Imaging and surgical reports were obtained to verify the diagnosis of spinal stenosis in all participants classified with SSS. Participants without previous spine imaging, reporting symptoms suggestive of spinal stenosis or progression of symptoms, were referred to spinal MRI at Oslo University Hospital.

#### Anthropometrics

Height and sitting-height were measured in centimetres (cm), using a wall-mounted measuring tape. Weight was measured in kilograms (kg) using a digital weight. Body mass index (BMI) was calculated as weight in kg divided by the height in meters squared (kg/m^2^). Arm span was measured (in cm) from a standing position with a non-stretchable tape, as the longest distance between the fingertips of the third fingers. Head circumference was measured (in cm) with a non-stretchable tape at the maximum diameter of the head.

### Assessment of physical functioning

The 6-min walk test (6MWT) was used to assess walking capacity. The 6MWT was conducted according to the American Thoracic Society Statement guidelines [26]. Ambulatory participants were instructed to walk as fast as possible, but not run, for 6 minutes in a 30-m corridor. Participants were allowed to use their usual walking aids if necessary. We recorded the total 6-min walking distance (6MWD) up to the nearest meter.

The electronic instrument Grippit was used to measure grip force and pinch grip [27]. Before the study began, the Grippit instrument was calibrated at Load Indicator System AB in Askim, Sweden. The test was conducted according to the “Basic Testing Procedure Using Grippit” [27]. The participants performed three trials with each hand, and alternated between the right and left hand. The best results for the maximum and average grip force and pinch grip registrations were recorded for each hand. The results were compared with age and gender matched reference values for Norwegian adults aged between 20 and 94 years [28]. For those eight participants aged between 16 to 19 years, we applied the reference values for the age group of 20 to 29 years, since differences are relatively small between these age groups [29].

To assess activities of daily living (ADL), we used the Stanford Health Assessment Questionnaire Disability Index (HAQ), a validated and widely used functional measure [30, 31]. HAQ consists of 20 questions in eight categories that represent a comprehensive set of functional activities: dressing and grooming, arising, eating, walking, hygiene, reach, grip and activities. Each item asks “Are you able to …” perform a particular task, and each category contains two or three activities [30]. In addition, participants are asked if they use assistive devices to perform the activities and if they need the assistance of another person. The response scale for each item is a four-level difficulty scale: 0 = without any difficulty, 1 = with some difficulty, 2 = with much difficulty, and 3 = unable to do [30].

Pain prevalence and intensity were measured using a pain drawing, and an 11-point Numeric Rating Scale (NRS) derived from the Norwegian Pain Society Minimum Questionnaire [32]. The participants were asked to mark their pain sites experienced the last week on the pain drawing. They also rated the maximum intensity of the most severe pain site on the NRS, with the range from 0 to 10 (0 = no pain, 10 = worst pain you can imagine) [33].

### Statistical analyses

Basic categorical demographic, anthropometric and clinical data are presented as frequencies and percentages. Continuous variables are presented as a mean with standard deviation (SD), or a median with range. Mean and SD are also used for some of the Likert scale variables (NRS and HAQ), assuming an equal gradual change between each category. For analysing differences between HAQ groups, we created a dichotomous variable of low difficulty (combining the HAQ categories 0 and 1) and high difficulty (combining the HAQ categories 2 and 3).

When estimating means and mean differences between groups, 10,000 percentile bootstrap replications were applied, with 95% confidence interval (CI) and *p*-value, as many of the variables seen here were not normally distributed. When analysing differences between the SSS and non-SSS group, a linear regression with bootstrap CI was applied for age adjustment, approximating the true group differences for dichotomous variables.

When analysing the median age of SSS onset, the directly observed median is not representative for the individual lifetime median age of onset, as our cross-sectional data tends to have a higher probability of including early than late SSS onset. Hence, SSS onset by age was estimated by: i) a logistic regression curve, using the observed SSS status at the time of inclusion in the study, and ii) a Kaplan-Meier estimation of SSS onset by age, censoring observation time at each patient’s attained age at the time of inclusion in the study.

SPSS version 25 (IBM Corp., Armonk, New York) and R version 3.6.1 (The R Foundation, Vienna, Austria) were used for the statistical analyses.

## Results

### Study population, demographic and anthropometric characteristics

**Fig. 1:**
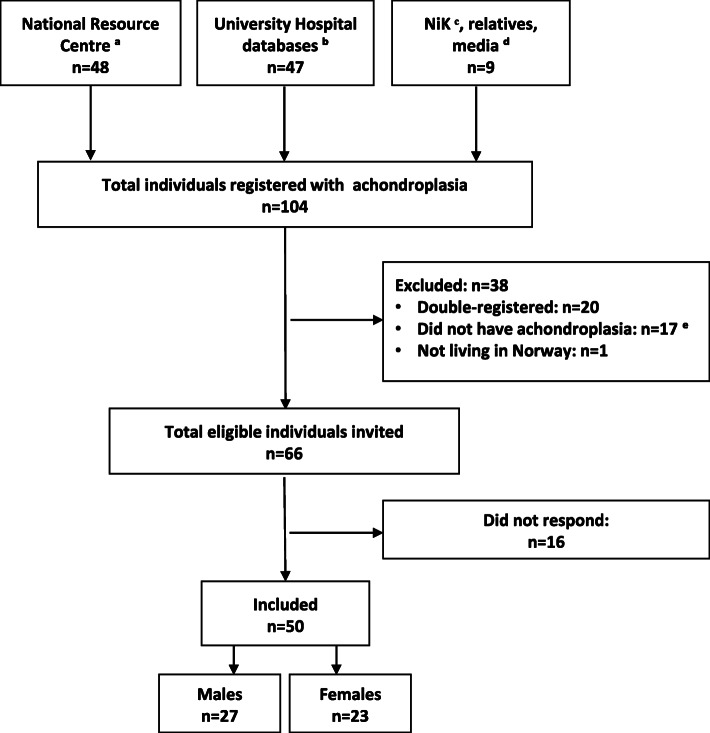
Flow of inclusion. ^a^ National Resource Centre for Rare Disorders, Sunnaas Rehabilitation Hospital. ^b^ The University Hospitals in Oslo, Bergen, Trondheim and Tromsø. ^c^ The Norwegian Restricted Growth Association (Norsk Interesseforening for Kortvokste). ^d^ You Tube, Facebook and Dagbladet Magasinet. ^e^ Had another or no skeletal dysplasia

From 66 identified adult individuals with achondroplasia living in Norway, we recruited 50 (76%), 27 males and 23 females. No participants meeting the inclusion criteria were excluded. Figure 1 details the recruitment of participants.

Achondroplasia was molecularly confirmed in all participants, all had the most commonly reported c.1138G > A (p.Gly380Arg) mutation in the *FGFR3*-gene [34]. The median age was 40.7 years (ranging from 16 to 87 years). Thirteen (26%) were married or cohabitants, 42% had completed university or college, 48% worked full-time or were students, and 30% received a full (≥90%) disability benefit (Table 1).

**Table 1:** Demographic characteristics in adults with achondroplasia (*n* = 50)

Characteristics	N (%)
Marital status	
Married or cohabitants	13 (26)
Single	30 (60)
Divorced, widower/widow	7 (14)
Education level, highest completed	
Compulsory school (≤10 years)	12 (24)
High school (11–13 years)	17 (34)
College or university	21 (42)
Employment status	
Working full time	13 (26)
Working part time (30–50%)	4 (8)
Student	11 (22)
Work rehabilitation	2 (4)
Age pension	5 (10)
Disability benefit, full (≥90%)	15 (30)
Main cause stenotic symptoms, n	11
Other causes, n	4

The mean height (SD) was 135.4 (9.5) cm for males, and 129.1 (7.6) cm for females. Arm span was 120.3 (8.6) cm and 110.6 (8.7) cm, respectively (Table 2).

**Table 2:** Anthropometric measurements of adults with achondroplasia

Variables	Males (*n* = 27)		Females (*n* = 23)	
	Mean (SD)	Range	Mean (SD)	Range
Height, cm	135.4 (9.5)	112.8–154.5	129.1 (7.6)	115.0–144.9
Weight, kg	62.4 (15.8)	42.1–95.8	54.0 (9.8)	32.3–68.6
Body mass index, kg/m^2^	34.0 (7.6)	21.7–49.9	32.4 (5.5)	21.8–43.8
Sitting height, cm ^a^	86.9 (4.6)	73.8–93.5	84.6 (4.0)	76.8–91.9
Arm span, cm	120.3 (8.6)	98.4–137.7	110.6 (8.7)	56.0–62.8
Head circumference, cm	60.4 (1.4)	57.1–63.0	59.1 (1.9)	56.0–62.8

^a^ Males: *n* = 25, females *n* = 22

### Findings of symptomatic spinal stenosis (SSS)

SSS was found in 34 of the 50 participants (68%), all with a central spinal stenosis (Table 3).

**Table 3:** Medical history of symptomatic spinal stenosis (SSS) in adults with achondroplasia (*n* = 34)

Age at symptom onset (years)	Affected spine level based on imaging reports	Narrowest AP spinal canal diameter (mm) ^a^	Total number of surgeries	Age at first surgery (years)	Time from symptom onset to first surgery (years)
10	T12-L2, L3-S1		2	10	0.3 ^b^
11	T12-L3		2	14	3
12	T11–12, L1-S1		1	22	10
12	T10–12, L2-S1		3	30	18
13	C3–7, T9–11, L1–5		1	25	12
13	C4–5, L1–5		2	17	4
14	L3–5		1	41	27
14	L1–3	L2–3: 7.9	0		
16	L2–4		1	30	14
16	L3–4	L3–4: 7.7	0		
17	T1–12, L1-S1		2	18	1
21	C3–4, L1–4		1	25	4
25	L1-S1		1	60	35
26	C4–5, L2-S1		1	28	2
26	T10–11, L2-S1		3	26	0.4 ^c^
27	T7–11	T7–8: 3.8	0		
29	C3–4, T8–12, L2–5		1	47	18
30	L3–4	L3–4: 8.0	0		
30	T12-L2		1	62	32
32	C4–6, L3–4		2	48	16
33	L2–5		1	45	12
34	T10-T12, L3–4		2	35	1
35	C4–7, L1–5		3	36	1
35	T8–11, L1–5		4	36	1
39	L3–5		1	46	7
41	T10–12, L1-S1		3	43	2
42	L2–4		1	44	2
43	C6-T1, T9–10, L3–5		1	63	20
43	L2–5		1	45	2
45	L2–5		1	53	8
46	C2, C4–5, C6–7, L1–3	L1–2: 5.9	0		
57	C3–4, L4–5	L1–2: 8.8	0		
60	C1-T1, L1–5		2	63	3
67	C3–5, L1–2		1	70	3

Abbreviations: C: Cervical, T: Thoracic, L: Lumbar

^a^ Narrowest anteroposterior (AP) spinal canal diameter assessed on sagittal MRI in non-operated individuals (*n* = 6) with SSS

^b^ 4 months

^c^ 5 months

In the non-SSS group (*n* = 16), all were asymptomatic. Eight had undergone spine MRI, of whom three were described with spinal stenosis by the imaging reports. As they were asymptomatic, they were classified as non-SSS. The remaining eight in the non-SSS group did not have an MRI.

Age at first symptom onset of spinal stenosis varied from 10 to 67 years (Table 3), including eight out of 34 reporting symptom onset before 16 years of age. According to the imaging reports, 32 out of 34 had two or more spinal levels affected, the lumbar spine being most frequently affected (Table 3 and Table 4).

**Table 4:** Clinical findings of symptomatic spinal stenosis in adults with achondroplasia (n = 34)

Characteristics		
Surgery for spinal stenosis, yes, n (%)		28 (82.4)
Median age at first surgery, years (range)		38.5 (10–70)
Mean time to first surgery, years (range) ^a^		9.2 (0.3–35)
Thoracolumbar kyphosis, n (%)		7 (20.6)
Abnormal neurological findings ^b^		N (%)
Tendon reflexes	Upper extremities	0 (0)
	Lower extremities	20 (58.8)
Sensation	Upper extremities	10 (29.4)
	Lower extremities	15 (44.1)
Muscle strength	Upper extremities	1 (2.9)
	Lower extremities	7 (20.6)
Urinary incontinence ^c^		14 (41.2)
Bowel incontinence ^c^		7 (20.6)

^a^ Time from symptom onset to first surgery

^b^ Abnormal neurological findings defined as:

Reflexes: 0, 3+ or clonus

Sensation: 0 or 1

Muscle strength: 0–3

^c^ As reported by participants

In the SSS group, 28 out of 34 participants had undergone at least one surgical operation for spinal stenosis. The majority experienced progressive symptoms of neurogenic claudication over months to years (Table 3), including decreasing walking distance. In addition, some had bladder and bowel symptoms. The median age at first spinal stenosis surgery was 38.5 years (range 10 to 70 years). The mean time from symptom onset to first surgery was 9.2 years, varying from 4 months to 35 years (Table 4). Two participants experienced a very rapid progression of symptoms within few weeks, necessitating acute decompression surgery. Six participants in the SSS group had not undergone spine surgery. These had mild to moderate reversible symptoms of neurogenic claudication, without progression. MRI confirmed spinal stenosis (Table 3). In the non-SSS group, none had a history of spinal stenosis surgery.

By clinical examination, 56% (19/34) in the SSS group had persistent abnormal neurological findings. Abnormal reflexes and reduced sensations in the lower extremities were the most common (Table 4). In addition, 41% (14/34) reported persistent urinary incontinence, and 21% (7/34) reported bowel incontinence. In the non-SSS group, none of the participants had abnormal neurological findings, urinary or bowel incontinence.

Seven participants (14%) had thoracolumbar kyphosis assessed by clinical examination, all in the SSS group.

### Estimated prevalence of symptomatic spinal stenosis (SSS) by age

**Fig. 2:**
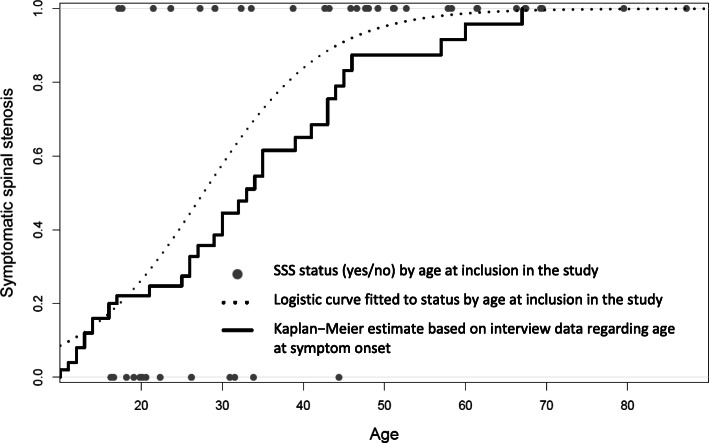
Symptomatic spinal stenosis (SSS) by age in adults with achondroplasia. The estimates are based on SSS status at time of inclusion in the study (dotted logistic regression curve) and interview data regarding age at symptom onset (drawn Kaplan-Meier curve). Based on the Kaplan-Meier plot, we estimate that 65% (95% confidence interval 44 to 78%) will have SSS by the age of 40, and 83% (95% confidence interval 62 to 93%) by the age of 45

Based on the reported age at symptom onset, the estimated median age for SSS was 33 years (95% CI 29 to 43 years). By the age of 40 years, about 65% (95% CI 44 to 78%) of individuals with achondroplasia will have SSS, and about 83% (95% CI 62 to 93%) will have SSS by the age of 45 years (Fig. 2).

### Physical functioning

In terms of walking capacity, 43 participants completed the 6MWT. Seven were unable to do the test, including the five visited at home, and an additional two due to other comorbidity. Of those seven, all but one had SSS. Maximum and average grip force and pinch grip were recorded, for each hand, in 45 participants (all except the five requiring a home visit). All participants completed the HAQ and pain assessments.

The mean (SD) age was 42.7 (20.0) years for the males and 38 (17.9) years for the females, with a mean difference of 4.7 (95% CI − 5.7 to 14.8, *p* = 0.39). We analysed the data for differences between males and females, but found no considerable differences regarding the 6MWT and the HAQ scores (Supplementary Table S1).

Males were stronger than females for all the absolute measurements (in Newton) for maximum grip force and pinch grip (Table S1). Maximum grip force was about 40%, and maximum pinch grip about 50%, compared with age and gender matched reference values for the Norwegian general population [28]. We found similar results for average grip force and pinch grip (data not shown).

The most frequent pain site locations were the back at 62% (31/50), the lower extremities at 42% (21/50), and the posterior neck at 14% (7/50). Regarding pain intensity, 38% (19/50) reported having had moderate pain (NRS 4–6) the last week, and 32% (16/50) reported severe pain (NRS 7–10) the last week. Mean pain intensity on a NRS scale (0–10) was significantly higher in females compared to males, with a mean difference of 1.9 (95% CI 0.2 to 3.6, *p* = 0.03) (Table S1).

The majority of the participants, 92% (46/50), reported the use of assistive devices. This included gripping aids (*n* = 26), jar openers (*n* = 17), tools with extended shafts (*n* = 15), walking aids, such as crutches, sticks, or walking frames (*n* = 11), manual (*n* = 12) or power (*n* = 22) wheelchairs, as well as stairs or stools (*n* = 25). Moreover, 16 out of 50 had a shower toilet at home, 9 out of 50 had height adjustable kitchen, and 14 out of 50 reported to have personal practical assistance at home.

### Comparison between individuals with and without symptomatic spinal stenosis

The SSS group was significantly older than the non-SSS group, with a mean difference of 24.5 years (95% CI 17.5 to 31.5 years, *p* < 0.01) (Table 5).

**Table 5:** Comparison between adults with achondroplasia with symptomatic spinal stenosis (SSS) and without (non-SSS)

Variables	SSS	Non-SSS	Unadjusted	Adjusted for age	
	(n = 34)	(n = 16)	Mean difference	Mean difference	*P* value
	Mean (SD)	Mean (SD)	(95% bootstrap CI)	(95% bootstrap CI)	
Age, mean, years	48.4 (17.6)	23.9 (7.8)	24.5 (17.5 to 31.5)	**–**	< 0.01
Gender males, % (n)	55.9 (15)	50.0 (8)	5.9 (−36.2 to 24.1)	3.5 (−34.9 to 42.6)	0.87
Body mass index, kg/m^2^	35.0 (6.8)	29.8 (5.0)	5.2 (1.9 to 8.5)	4.0 (−0.2 to 7.9)	0.05
Employment, % (n) ^a^	26.5 (9)	93.8 (15)	−67.3 (−84.8 to 45.8)	−33.7 (−62.3 to −7.2)	0.03
Wheelchair users, % (n) ^b^	52.9 (18)	25 (4)	27.9 (−0.6 to 54.7)	51.2 (19.8 to 76.9)	< 0.01
**Physical Functioning**					
6MWT, meters ^c^	386 (129)	526 (59)	− 140 (− 196 to − 85)	−110 (−172 to −40)	< 0.01
Grip force, maximum ^d^					
Right hand, Newton	162.5 (71.5)	170.9 (61.8)	−8.3 (−47.9 to 30.2)	−26.5 (−64.8 to 12.1)	0.17
Left hand, Newton	155.1 (69.2)	148.1 (43.3)	7.0 (−25.6 to 39.5)	−9.6 (− 42.6 to 29.6)	0.59
Pinch grip, maximum ^d^					
Right hand, Newton	35.2 (9.5)	33.8 (10.1)	1.4 (−4.6 to 7.4)	−3.8 (−10.8 to 2.8)	0.27
Left hand, Newton	34.3 (12.6)	35.4 (10.2)	−1.2 (−7.9 to 5.6)	−7.2 (− 15.2 to 1.5)	0.09
HAQ Total mean score ^e^	1.1 (0.7)	0.3 (0.2)	0.8 (0.6 to 1.1)	0.3 (0.06 to 0.6)	0.04
HAQ Category sum scores ^e^					
Dressing and grooming	1.0 (0.8)	0.1 (0.3)	0.9 (0.6 to 1.2)	0.4 (0.02 to 0.8)	0.04
Arising	0.9 (1.0)	0.1 (0.3)	0.8 (0.4 to 1.2)	0.1 (−0.3 to 0.4)	0.76
Eating	0.6 (0.9)	0.5 (0.6)	0.1 (−0.3 to 0.5)	− 0.3 (− 0.9 to 0.2)	0.38
Walking	1.6 (0.9)	0.3 (0.5)	1.2 (0.9 to 1.6)	0.9 (0.3 to 1.4)	< 0.01
Hygiene	1.4 (0.8)	0.3 (0.5)	1.1 (0.7 to 1.5)	0.6 (0.2 to 1.0)	< 0.01
Reach	1.2 (0.9)	0.1 (0.3)	1.1 (0.7 to 1.4)	0.4 (−0.06 to 0.8)	0.07
Grip	0.5 (0.9)	0.0 (0.0)	0.5 (0.3 to 0.8)	0.2 (−0.3 to 0.6)	0.49
Activities	1.8 (0.9)	0.9 (0.3)	0.9 (0.6 to 1.2)	0.4 (−0.01 to 0.8)	0.08
**Pain intensity**, NRS, mean ^f^	5.4 (3.1)	3.5 (3.1)	1.9 (0.06 to 3.8)	3.2 (0.6 to 5.6)	0.02

^a^ Working full-time or student

^b^ Including both permanent and sporadic wheelchair users, and manual or power wheelchairs

^c^ 6-min walk test: SSS group *n* = 28, non-SSS group: n = 15

^d^ Grip force and pinch grip: SSS group: *n* = 29, non-SSS group: n = 16

^e^ Health Assessment Questionnaire, score 0–3

^f^ Numeric Rating Scale 0–10 (best to worst)

In the SSS group, 26% (9/34) were employed (working full-time or students), compared with 94% (15/16) in the non-SSS group (*p* = 0.03). Mean walking distance (6MWD) was 110 m shorter for individuals in the SSS group compared with those in the non-SSS group (95% CI − 172 to − 40 m, p < 0.01) (Table 5). For grip force and pinch grip, there were no considerable differences between the two groups (Table 5).

**Fig. 3:**
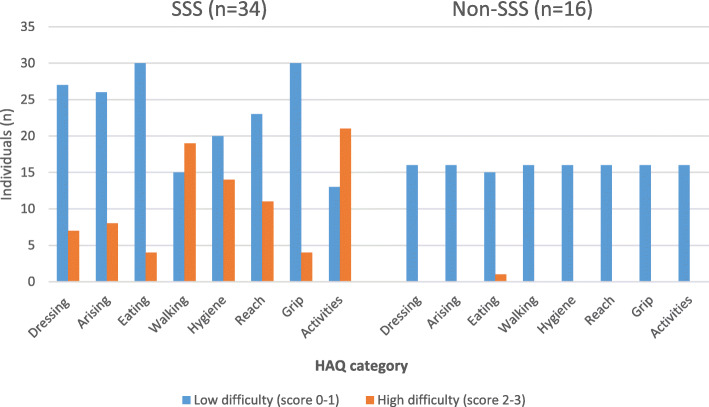
Function in activities of daily living (ADL) in adults with achondroplasia assessed by the Health Assessment Questionnaire (HAQ) for individuals with symptomatic spinal stenosis (SSS) and without (non-SSS). The number of individuals reporting high difficulty score (much difficulty or not able to) was higher in the SSS group for all the HAQ categories compared with the non-SSS group, reflecting more limitations in ADL

Individuals with SSS reported higher HAQ scores for both total mean HAQ and for all the eight subcategories, reflecting more activity limitations (Table 5 and Fig. 3). The categories *walking* and *activities* had the highest total HAQ scores for individuals with SSS, at 1.6 (SD 0.9) and 1.8 (SD 0.9), respectively. After adjusting for age, the differences for the total mean HAQ, and the categories *dressing and grooming, walking*, and *hygiene* remained statistically significant. In the SSS group, 47% (16/34) reported the use of ambulatory aids (crutches, sticks, or walking frames) compared with 0/16 in the non-SSS group. Regarding the use of wheelchairs, 53% (18/34) in SSS group reported to be a wheelchair user, compared with 25% (4/16) in the non-SSS group (Table 5). Only three were permanent wheelchair users (all with SSS).

Mean pain intensity (NRS 0–10) was significantly higher in the SSS group compared with the non-SSS group, with a mean difference of 3.2 (95% CI 0.6 to 5.6, *p* = 0.02).

### Other neurosurgical and orthopaedic treatment history

We looked for differences between the SSS and non-SSS group in terms of other neurosurgical or orthopaedic complications, including prevalence of arthritis in major weight-bearing joints, but did not find any clinically relevant differences (Table 6). Statistical analyses were not applicable, due to the small numbers.

**Table 6:** Other neurosurgical and orthopaedic complications in adults with achondroplasia with symptomatic spinal stenosis (SSS) and without (non-SSS)

Complications	SSS	Non-SSS
	(n = 34)	(n = 16)
	N (%)	N (%)
**Past neurosurgical treatment**		
VP-shunt or ventriculostomy ^a^	6 (17.6)	1 (6.3)
Foramen magnum decompression	1 (2.9)	1 (6.3)
**Past orthopaedic treatment**		
Surgery for kyphosis ^b^	1 (2.9)	0 (0)
Tibia osteotomy	9 (26.5)	6 (37.5)
With lower limb lengthening	8 (23.5)	3 (18.8)
Humeral lengthening	1 (2.9)	2 (12.5)
Hip arthritis	2 (5.9)	0 (0)
Knee arthritis	2 (5.9)	0 (0)
Lateral meniscus rupture	1 (2.9)	4 (25.0)

^a^*VP-shunt* Ventriculo-peritoneal shunt

^b^ Thoracolumbar kyphosis

## Discussion

We found a very high prevalence of SSS in adults with achondroplasia in this study. The majority had symptom onset at a young age with multiple spinal levels affected. The presence of SSS was associated with reduced walking capacity, activity limitations, and moderate or high levels of pain.

The prevalence of SSS in our study is much higher than reported in commonly cited studies of achondroplasia [10–13], but consistent with a recently published study from Japan [18]. However, none of these studies have described how SSS was defined. The high proportion of SSS in our study might be explained by the study population’s higher mean age compared with previous studies, as the prevalence of SSS is known to increase with age [6, 11, 35]. Another explanation might be our study’s clinical approach, as we specifically asked about and clinically examined for symptoms of spinal stenosis in all participants.

Several studies have reported a clear association between thoracolumbar kyphosis and the development of SSS [4, 9, 36]. In our study, 14% of the participants had kyphosis, all with SSS. In a large US study, more than 50% of the participants with achondroplasia had moderate to severe kyphosis [37]. The study was conducted at a tertiary referral centre, and used radiologic assessment of kyphosis, which might explain some of the difference to our numbers. Other studies have reported a prevalence of 10 to 15% [38].

There are currently no widely accepted quantitative criteria for the diagnosis of spinal stenosis in the general population or in individuals with achondroplasia [6, 39, 40]. In addition there is a poor correlation between stenosis on imaging and patient’s symptoms [5, 6]. In many studies, surgery has been regarded as the gold standard for the assessment of spinal stenosis [41]. According to the guidelines of the North American Spine Society and other authors, we applied a combination of characteristic symptoms and spinal stenosis described in the imaging reports to establish the diagnosis of SSS [6, 21, 41]. In addition, spinal stenosis was confirmed by surgical records in 28 participants who had undergone stenosis surgery.

The early onset of SSS in this study is consistent with other studies [7–9, 18], but is in marked contrast to the average statured population, where symptoms rarely present before 60 years of age [6]. Healthcare professionals managing individuals with achondroplasia must be aware of this, so as not to overlook or neglect symptoms of spinal stenosis.

In our study, the mean time from the first symptom onset to spinal stenosis surgery was 9.2 years, which is in contrast to more recent publications recommending an early surgical intervention [2, 35, 42]. We found a marked variability in the clinical presentation and progression of SSS, in line with Pyeritz et al. [9]. The majority of participants reported gradually progressive, but reversible symptoms of neurogenic claudication. Some individuals had slow or almost no progression in clinical symptoms over years, while other had a very rapid progression within few weeks. The findings underline the importance of thorough assessment and monitoring of SSS in achondroplasia.

Our findings of multilevel spinal stenosis in achondroplasia have also been reported in other studies [3, 7, 9]. This is in contrast to the average statured population, where the lumbar spine is primarily affected [5, 39]. These findings underline the importance of performing imaging of the entire spine in achondroplasia, preferably by MRI, in the presence of clinical symptoms suggestive of spinal stenosis [5, 7].

Walking is important in everyday activities and for participation. The 6MWT reflects functional walking capacity at a similar level as required for daily activities [43]. In a systematic review, Bohannon et al. (2016) found that a difference of 30 m or more in 6MWD was of clinical importance [44]. The 110-m shorter 6MWD in the SSS group was not explained by differences in orthopaedic complications of the lower extremities between the two groups. Moreover, very few participants reported arthritis in major weight-bearing joints, consistent with the findings of Lee et al. [45].

The high proportion of the SSS group who used walking aids or wheelchairs reflects some of the practical consequences of reduced walking capacity in this group. Many were able to walk indoors or for short distances without any helping aids, but needed walking aids or wheelchairs for longer distances, such as going to the shopping centre or travelling. The high HAQ mean score for the SSS group in the category *activities* reflects these limitations in tasks such as running errands and shopping, and is consistent with the findings of Alade et al. [17].

In this study, we used the complementary HAQ scoring manual (the Alternative Disability Index), which does not give additional scores for using assistive devices when performing the tasks [30]. We considered that this better reflected the real-life situations of the participants with achondroplasia, since the majority reported using assistive devices or compensating strategies when performing ADL. These strategies also included climbing stairs and stools (for instance, in the kitchen, bathroom, and the grocery store), but the majority did not consider this to be a problem. However, after the onset of SSS, these strategies were reported to be markedly limited or impossible. This is consistent with the findings of Matsushita et al., who observed that lower physical health scores were strongly associated with a history of spine surgery [18]. Also the HAQ scores for the *walking* and *hygiene* categories reflect this, showing the most significant differences between the SSS and non-SSS group. The high prevalence of persistent urinary and bowel incontinence in the SSS group might have considerable negative social impact, limiting daily activities, productivity at work, and social activities [46].

There were no differences in hand strength between the SSS and non-SSS group. The clinical neurological examination supported these findings, since few participants had abnormal neurological findings in their upper extremities. In addition, no considerable differences were found between the two groups for the HAQ *grip* category. Interestingly, compared with age and gender matched reference values, the achondroplasia study population scored significantly lower (about 40–50%) for all parameters related to both grip force and pinch grip. Lower body height might explain some of these differences [28], but these findings could also reflect isolated reduced muscle strength within the achondroplasia population, as suggested by Sims et al. [47].

In this study, 70% of the participants reported having had moderate to severe pain the last week. This is consistent with previous studies, which reported a pain prevalence of 64–75% in adults with achondroplasia [15–17]. Pain intensity was significantly higher in the SSS group compared with the non-SSS group. Females reported more pain than males, consistent with the findings of Alade et al. [17]. This high prevalence of pain might have an impact on daily functioning, including social and family life, and the ability to work [33].

The education level in the study population was equivalent to or higher than in the general Norwegian population [48]. Despite this, only one third of the SSS group were currently employed, compared with 94% of the non-SSS group. Of those not employed, the majority (73%) reported SSS as the main cause. This is consistent with the findings of Ain et al., who found a negative effect of back or leg pain on work participation, and a marked progression within 1-year of follow-up in adults with achondroplasia [35].

### Clinical implications and further research

The high prevalence, early symptom onset, and multilevel spinal affection, underline the importance of thorough assessment and management of SSS in individuals with achondroplasia. Evidence indicates that individuals with achondroplasia might benefit from early surgical intervention [2], and several authors have recommended that individuals presenting with symptoms of spinal stenosis should seek medical advice as soon as possible, in order to avoid permanent spinal cord injury [2, 9, 18, 35, 42].

The cross-sectional design of our study does not answer the question of whether some of the associated negative consequences of SSS on physical functioning could have been prevented through improved management or earlier surgical intervention. This needs to be explored in future longitudinal studies.

### Strengths and limitations

The high response rate and broad recruitment of participants, all with genetically confirmed achondroplasia, constituted major strengths of this study. Furthermore, the clinical study design, face-to-face interview with each participant, objective measurement methods, and minimal missing data are other factors that strengthen our findings.

There are, however, several limitations to this study. First, as there is no central register, the exact prevalence of achondroplasia within Norway is uncertain. In a recent large population-based study, the prevalence of achondroplasia in Europe was found to be 3.1 per 100,000 live births [49]. According to Statistics Norway, there were about 50,000 to 60,000 annual live births in Norway during the relevant period up to the year 2002 [50], meaning that approximately 1.7 new achondroplasia infants were born each year. A 10-year shorter lifespan has been reported for adults with achondroplasia in the US, due to different medical complications and accidents [51]. We do not have reason to believe that this is different in Norway. The expected population of adults (16 years of age or older) with achondroplasia in Norway is therefore estimated to be between 66 and 101 adults. We recruited somewhat fewer individuals for this study, which could risk selection bias. Second, the preoperative images were not available for all the participants, since many had undergone their first stenosis surgery several decades ago. However, imaging and surgical records were obtained for all classified with SSS. Third, recall bias could potentially influence the medical history. Medical records were obtained to confirm the medical information and reduce this risk. Fourth, the instruments used to assess physical functioning in this study have not been validated for achondroplasia, but are commonly used in clinical practice for a variety of conditions [26, 27, 30, 43], including spinal stenosis [52].

## Conclusions

This study found a very high prevalence of SSS among Norwegian adults with achondroplasia, which we believe is representative of this population worldwide. The majority had symptom onset at a young age, and with multiple spinal levels affected. The presence of SSS was associated with reduced walking distance, a higher degree of activity limitations, and more pain than those without this complication. The findings underline the importance of thorough assessment and monitoring of SSS in achondroplasia, including a formal assessment of physical functioning.

## Supplementary information

**Additional file 1.** Physical functioning in adult males and females with achondroplasia (*n*=50).

## Data Availability

The data that support the findings of this study are not publicly available because of the potential of identifying individual participants. In case of a specific scientific question, requests can be addressed to the corresponding author.

## References

[1] Ornitz DM, Legeai-Mallet L (2017). Achondroplasia: development, pathogenesis, and therapy. Dev Dynamics.

[2] Carlisle ES, Ting BL, Abdullah MA, Skolasky RL, Schkrohowsky JG, Yost MT (2011). Laminectomy in patients with achondroplasia: the impact of time to surgery on long-term function. Spine..

[3] Schkrohowsky JG, Hoernschemeyer DG, Carson BS, Ain MC (2007). Early presentation of spinal stenosis in achondroplasia. J Pediatr Orthop.

[4] Pauli RM (2019). Achondroplasia: a comprehensive clinical review. Orphanet J Rare Dis.

[5] Kreiner DS, Shaffer WO, Baisden JL, Gilbert TJ, Summers JT, Toton JF (2013). An evidence-based clinical guideline for the diagnosis and treatment of degenerative lumbar spinal stenosis (update). Spine J.

[6] Suri P, Rainville J, Kalichman L, Katz JN (2010). Does this older adult with lower extremity pain have the clinical syndrome of lumbar spinal stenosis?. Jama..

[7] Sciubba DM, Noggle JC, Marupudi NI, Bagley CA, Bookland MJ, Carson BS (2007). Spinal stenosis surgery in pediatric patients with achondroplasia. J Neurosurg.

[8] Thomeer RT, van Dijk JM (2002). Surgical treatment of lumbar stenosis in achondroplasia. J Neurosurg.

[9] Pyeritz RE, Sack GH, Udvarhelyi GB (1987). Thoracolumbosacral laminectomy in achondroplasia: long-term results in 22 patients. Am J Med Genet.

[10] Unger S, Bonafe L, Gouze E (2017). Current care and investigational therapies in achondroplasia. Curr Osteoporos Rep.

[11] Hunter AG, Bankier A, Rogers JG, Sillence D, Scott CI (1998). Medical complications of achondroplasia: a multicentre patient review. J Med Genet.

[12] Hall JG (1988). The natural history of achondroplasia. Basic Life Sci.

[13] Wright MJ, Irving MD (2012). Clinical management of achondroplasia. Arch Dis Child.

[14] Fredwall SO, Maanum G, Johansen H, Snekkevik H, Savarirayan R, Lidal IB (2020). Current knowledge of medical complications in adults with achondroplasia: a scoping review. Clin Genet.

[15] Jennings SE, Ditro CP, Bober MB, Mackenzie WG, Rogers KJ, Conway L (2019). Prevalence of mental health conditions and pain in adults with skeletal dysplasia. Qual Life Res Int J Qual Life Asp Treat Care Rehab.

[16] Dhiman N, Albaghdadi A, Zogg CK, Sharma M, Hoover-Fong JE, Ain MC (2017). Factors associated with health-related quality of life (HRQOL) in adults with short stature skeletal dysplasias. Qual Life Res Int J Qual Life Asp Treat Care Rehab.

[17] Alade Y, Tunkel D, Schulze K, McGready J, Jallo G, Ain M (2013). Cross-sectional assessment of pain and physical function in skeletal dysplasia patients. Clin Genet.

[18] Matsushita M, Kitoh H, Mishima K, Yamashita S, Haga N, Fujiwara S (2019). Physical, mental, and social problems of adolescent and adult patients with achondroplasia. Calcif Tissue Int.

[19] von Elm E, Altman DG, Egger M, Pocock SJ, Gotzsche PC, Vandenbroucke JP (2014). The strengthening the reporting of observational studies in epidemiology (STROBE) statement: guidelines for reporting observational studies. Int J Surg.

[20] TRS National Resource Centre for Rare Disorders. https://www.sunnaas.no/trs. Accessed 10 Jan 2020.

[21] Katz JN, Harris MB (2008). Clinical practice. Lumbar spinal stenosis. N Engl J Med.

[22] Verbiest H (1975). Pathomorphologic aspects of developmental lumbar stenosis. Orthop Clin North Am.

[23] Steurer J, Roner S, Gnannt R, Hodler J (2011). Quantitative radiologic criteria for the diagnosis of lumbar spinal stenosis: a systematic literature review. BMC Musculoskelet Disord.

[24] Kalichman L, Cole R, Kim DH, Li L, Suri P, Guermazi A (2009). Spinal stenosis prevalence and association with symptoms: the Framingham study. Spine J.

[25] Buckup J. Clinical test for the musculoskeletal system. 3rd ed. Stuttgart: Thieme; 2016.

[26] American Thoracic Society statement: guidelines for the six-minute walk test. Am J Respir Crit Care Med. 2002;166(1):111–7. 10.1164/ajrccm.166.1.at1102 12091180

[27] Nordenskiold UM, Grimby G (1993). Grip force in patients with rheumatoid arthritis and fibromyalgia and in healthy subjects. A study with the Grippit instrument. Scand J Rheumatol.

[28] Nilsen T, Hermann M, Eriksen CS, Dagfinrud H, Mowinckel P, Kjeken I (2012). Grip force and pinch grip in an adult population: reference values and factors associated with grip force. Scand J Occup Ther.

[29] Hager-Ross C, Rosblad B (2002). Norms for grip strength in children aged 4–16 years. Acta Paediatrica (Oslo, Norway : 1992).

[30] Bruce B, Fries JF (2003). The Stanford health assessment questionnaire: a review of its history, issues, progress, and documentation. J Rheumatol.

[31] White DK, Wilson JC, Keysor JJ (2011). Measures of adult general functional status: SF-36 physical functioning subscale (PF-10), health assessment questionnaire (HAQ), modified health assessment questionnaire (MHAQ), Katz index of Independence in activities of daily living, functional Independence measure (FIM), and osteoarthritis-function-computer adaptive test (OA-function-CAT). Arthritis Care Res.

[32] Fredheim OM, Borchgrevink PC, Landmark T, Schjodt B, Breivik H (2008). A new schedule for the inventory of pain. Tidsskr Nor Laegeforen.

[33] Breivik H, Borchgrevink PC, Allen SM, Rosseland LA, Romundstad L, Hals EK (2008). Assessment of pain. Br J Anaesth.

[34] Vajo Z, Francomano CA, Wilkin DJ (2000). The molecular and genetic basis of fibroblast growth factor receptor 3 disorders: the achondroplasia family of skeletal dysplasias, Muenke craniosynostosis, and Crouzon syndrome with acanthosis nigricans. Endocr Rev.

[35] Ain MC, Abdullah MA, Ting BL, Skolasky RL, Carlisle ES, Schkrohowsky JG (2010). Progression of low back and lower extremity pain in a cohort of patients with achondroplasia. J Neurosurg Spine.

[36] Huet T, Cohen-Solal M, Laredo JD, Collet C, Baujat G, Cormier-Daire V (2020). Lumbar spinal stenosis and disc alterations affect the upper lumbar spine in adults with achondroplasia. Sci Rep.

[37] Khan BI, Yost MT, Badkoobehi H, Ain MC (2016). Prevalence of scoliosis and thoracolumbar kyphosis in patients with achondroplasia. Spine Deformity.

[38] Pauli RM, Breed A, Horton VK, Glinski LP, Reiser CA (1997). Prevention of fixed, angular kyphosis in achondroplasia. J Pediatr Orthop.

[39] Schroeder GD, Kurd MF, Vaccaro AR (2016). Lumbar spinal stenosis: how is it classified?. J Am Acad Orthop Surg.

[40] Jeong ST, Song HR, Keny SM, Telang SS, Suh SW, Hong SJ (2006). MRI study of the lumbar spine in achondroplasia. A morphometric analysis for the evaluation of stenosis of the canal. J Bone Joint Surg.

[41] North American Spine Society. Diagnosis and treatment of degenerative lumbar spinal stenosis 2011. www.spine.org/Research-Clinical-Care/Quality-Improvement/ClinicalGuidelines. Accessed Sep 2019.

[42] Bodensteiner JB (2019). Neurological manifestations of achondroplasia. Curr Neurol Neurosci Rep.

[43] Bennell K, Dobson F, Hinman R (2011). Measures of physical performance assessments: self-paced walk test (SPWT), stair climb test (SCT), six-minute walk test (6MWT), chair stand test (CST), timed up & go (TUG), sock test, lift and carry test (LCT), and car task. Arthritis Care Res.

[44] Bohannon RW, Crouch R (2017). Minimal clinically important difference for change in 6-minute walk test distance of adults with pathology: a systematic review. J Eval Clin Pract.

[45] Lee ST, Song HR, Mahajan R, Makwana V, Suh SW, Lee SH (2007). Development of genu varum in achondroplasia: relation to fibular overgrowth. J Bone Joint Surg.

[46] Landefeld C. Seth (2008). National Institutes of Health State-of-the-Science Conference Statement: Prevention of Fecal and Urinary Incontinence in Adults. Annals of Internal Medicine.

[47] Sims DT, Onambele-Pearson GL, Burden A, Payton C, Morse CI (2018). Specific force of the vastus lateralis in adults with achondroplasia. J Appl Physiol (1985).

[48] Statistics Norway. Educational attainment of the population. https://www.ssb.no/en/utdanning/statistikker/utniv/aar. Accessed 10 Jan 2020.

[49] Coi A, Santoro M, Garne E, Pierini A, Addor MC, Alessandri JL (2019). Epidemiology of achondroplasia: a population-based study in Europe. Am J Med Genet A.

[50] Statistics Norway. Facts about the population (Fakta om befolkningen) https://www.ssb.no/befolkning/faktaside/befolkningen. Accessed 10 Jan 2020.

[51] Wynn J, King TM, Gambello MJ, Waller DK, Hecht JT (2007). Mortality in achondroplasia study: a 42-year follow-up. Am J Med Genet A.

[52] Forsth P, Olafsson G, Carlsson T, Frost A, Borgstrom F, Fritzell P (2016). A randomized, controlled trial of fusion surgery for lumbar spinal stenosis. N Engl J Med.

