# Correction to: High prevalence of symptomatic spinal stenosis in Norwegian adults with achondroplasia: a population-based study

**DOI:** 10.1186/s13023-020-01636-w

**Published:** 2020-12-07

**Authors:** Svein O. Fredwall, Unni Steen, Olga de Vries, Cecilie F. Rustad, Heidi Beate Eggesbø, Harald Weedon-Fekjær, Ingeborg B. Lidal, Ravi Savarirayan, Grethe Månum

**Affiliations:** 1 TRS National Resource Centre for Rare Disorders, Sunnaas Rehabilitation Hospital, Nesodden, Norway; 2 Faculty of Medicine, Institute of Clinical Medicine, University of Oslo, Oslo, Norway; 3 Department of Medical Genetics, Oslo University Hospital, Oslo, Norway; 4 Division of Radiology and Nuclear Medicine, Oslo University Hospital, Oslo, Norway; 5 Oslo Centre for Biostatistics and Epidemiology, Research Support Service, Oslo University Hospital, Oslo, Norway; 6 Murdoch Children’s Research Institute, University of Melbourne, Parkville, Australia; 7 Department of Research, Sunnaas Rehabilitation Hospital, Nesodden, Norway

## Correction to: Orphanet Journal of Rare Diseases (2020) 15:123 10.1186/s13023-020-01397-6

Following the publication of the original article [1], the authors became aware of the following error in Table 2:

In females, the correct range for arm span should be 97.6–134.9, instead of 56.0–62.8 as presented in the original article.

The corrected Table 2 is shown here below:

**Table 2:** Anthropometric measurements of adults with achondroplasia

Variables	Males (n = 27)		Females (n=23)	
	Mean (SD)	Range	Mean (SD)	Range
Height, cm	135.4 (9.5)	112.8–154.5	129.1 (7.6)	115.0–144.9
Weight, kg	62.4 (15.8)	42.1–95.8	54.0 (9.8)	32.3–68.6
Body mass index, kg/m^2^	34.0 (7.6)	21.7–49.9	32.4 (5.5)	21.8–43.8
Sitting height, cm^a^	86.9 (4.6)	73.8–93.5	84.6 (4.0)	76.8–91.9
Arm span, cm	120.3 (8.6)	98.4–137.7	110.6 (8.7)	97.6–134.9
Head circumference, cm	60.4 (1.4)	57.1–63.0	59.1 (1.9)	56.0–62.8

^a^Males: n = 25, females n = 22

## References

[1] Fredwall SO (2020). High prevalence of symptomatic spinal stenosis in Norwegian adults with achondroplasia: a population-based study. Orphanet J Rare Dis.

