# Supplementary material for: High prevalence of symptomatic spinal stenosis in Norwegian adults with achondroplasia: a population-based study
Source: Orphanet J Rare Dis. 2020 May 25;15:123. doi: 10.1186/s13023-020-01397-6 (PMC7249360; doi:10.1186/s13023-020-01397-6)
Supplement: Supplementary file 1 — Additional file 1. Physical functioning in adult males and females with achondroplasia (n=50). [file 13023_2020_1397_MOESM1_ESM.docx]

**Table S1** Physical functioning in adult males and females with achondroplasia (n=50).

| Variables | Males (n=27)  Mean (SD) | Females (n=23)  Mean (SD) | Mean difference  (95% bootstrap CI) | P value |
| --- | --- | --- | --- | --- |
| 6MWT, meters ^a^ | 422 (150) | 447 (105) | -25 (-106 to 48) | 0.53 |
| Grip force, maximum ^b^ |  |  |  |  |
| Right hand: Newton | 206.1 (63.0) | 123.0 (41.5) | 83.1 (52.8 to 114.5) | <0.01 |
| % of norm ^c^ | 40.2 | 39.2 |  |  |
| Left hand: Newton | 190.5 (53.7) | 113.1 (39.1) | 77.4 (50.7 to 105.5) | <0.01 |
| % of norm ^c^ | 38.9 | 38.1 |  |  |
| Pinch grip, maximum ^b^ |  |  |  |  |
| Right hand: Newton | 39.4 (9.0) | 29.7 (7.7) | 9.8 (5.0 to 14.6) | <0.01 |
| % of norm ^c^ | 49.1 | 54.7 |  |  |
| Left hand: Newton | 41.2 (10.2) | 27.9 (9.1) | 13.3 (7.6 to 18.9) | <0.01 |
| % of norm ^c^ | 51.1 | 53.0 |  |  |
| HAQ Total mean score ^c^ | 0.8 (0.7) | 0.9 (0.7) | -0.03 (-0.4 to 0.4) | 0.87 |
| HAQ Category sum scores ^d^ |  |  |  |  |
| Dressing and grooming | 0.8 (0.8) | 0.7 (0.9) | 0.08 (-0.4 to 0.5) | 0.72 |
| Arising | 0.6 (1.0) | 0.6 (0.9) | -0.02 (-0.5 to 0.5) | 0.95 |
| Eating | 0.5 (0.8) | 0.7 (0.8) | -0.1 (-0.6 to 0.3) | 0.55 |
| Walking | 1.2 (1.0) | 1.1 (0.8) | 0.1 (-0.4 to 0.7) | 0.62 |
| Hygiene | 1.1 (0.9) | 1.1 (0.9) | -0.01 (-0.5 to 0.5) | 0.96 |
| Reach | 0.9 (1.0) | 0.8 (0.9) | 0.06 (-0.5 to 0.6) | 0.82 |
| Grip | 0.2 (0.6) | 0.5 (0.9) | -0.3 (-0.7 to 0.1) | 0.17 |
| Activities | 1.4 (0.9) | 1.5 (0.8) | -0.08 (-0.5 to 0.4) | 0.75 |
| Pain intensity, NRS, mean ^e^ | 3.9 (3.1) | 5.9 (3.1) | -1.9 (-3.6 to -0.2) | 0.03 |

^a^ 6-Minute walk test; males n=21, females n=22

^b^ Grip force and pinch grip: males n=23, females n=22

^c^ Norm for average-statured Norwegian individuals based on age and gender

^d^ Health Assessment Questionnaire, score 0-3

^e^ Numeric Rating Scale 0-10 (best to worst)
